# Genomic Characterisation of Three Mapputta Group Viruses, a Serogroup of Australian and Papua New Guinean Bunyaviruses Associated with Human Disease

**DOI:** 10.1371/journal.pone.0116561

**Published:** 2015-01-14

**Authors:** Penelope J. Gauci, Jane McAllister, Ian R. Mitchell, David B. Boyle, Dieter M. Bulach, Richard P. Weir, Lorna F. Melville, Aneta J. Gubala

**Affiliations:** 1 Land Division, Defence Science & Technology Organisation, Fishermans Bend, Victoria, Australia; 2 Australian Animal Health Laboratory, Commonwealth Scientific and Industrial Research Organisation, Geelong, Victoria, Australia; 3 Berrimah Veterinary Laboratories, Department of Primary Industry and Fisheries, Berrimah, Northern Territory, Australia; Metabiota, UNITED STATES

## Abstract

The Mapputta serogroup tentatively contains the mosquito-associated viruses Mapputta, Maprik, Trubanaman and Gan Gan. Interestingly, this serogroup has previously been associated with an acute epidemic polyarthritis-like illness in humans; however, there has been no ensuing genetic characterisation. Here we report the complete genome sequences of Mapputta and Maprik viruses, and a new Mapputta group candidate, Buffalo Creek virus, previously isolated from mosquitoes and detected by serology in a hospitalised patient. Phylogenetic analyses indicate that the group is one of the earliest diverged groups within the genus *Orthobunyavirus* of the family *Bunyaviridae*. Analyses show that these three viruses are related to the recently sequenced Australian bunyaviruses from mosquitoes, Salt Ash and Murrumbidgee. A notable feature of the Mapputta group viruses is the absence of the NSs (non-structural) ORF commonly found on the S segment of other orthobunyaviruses. Viruses of the Mapputta group have been isolated from geographically diverse regions ranging from tropical Papua New Guinea to the semi-arid climate of south-eastern Australia. The relevance of this group to human health in the region merits further investigation.

## Introduction

The family *Bunyaviridae* consists of more than 350 assigned viruses, making it one of the largest taxonomic groupings of RNA viruses [1]. Viruses in this family have a tripartite, negative sense, single-stranded RNA genome. The large (L) segment encodes the RNA dependent RNA polymerase, the medium (M) segment encodes the glycoproteins Gn and Gc and the small (S) segment encodes the nucleoprotein. The family comprises five genera; *Hantavirus, Orthobunyavirus, Phlebovirus, Nairovirus*, and *Tospovirus*, separated on the basis of serological and molecular characteristics [1]. Most bunyaviruses are arthropod-transmitted, with the exception of those in the genus *Hantavirus*, which are rodent-borne. Genus *Orthobunyavirus* is the largest and most complex of the five genera and is currently represented by more than 170 viruses, with 48 assigned species and 18 serogroups [1]. The size pattern of the genomic RNA segments, the size of the viral structural proteins and consensus of the viral RNA 3’ and 5’ termini, can be used to distinguish orthobunyaviruses from other genera. In addition to the structural proteins encoded by the genome, the M segment of orthobunyaviruses also encodes a non-structural protein, NSm, and in most cases the S segment encodes a non-structural protein, NSs [2].

The Mapputta serogroup of viruses contains the antigenically cross-reactive viruses Mapputta (MAPV), Maprik (MPKV), Trubanaman (TRUV) and Gan Gan (GGV) [1]. It remains an unassigned group in the family *Bunyaviridae* due to the lack of further biochemical characterisation. The group is suggested to belong to the genus *Orthobunyavirus* based on the presumed transmissibility by mosquitoes, and molecular weights of specific viral proteins and the genomic RNA segments [3]. The first isolation of MAPV was in 1960, from a pool of engorged *Anopheles meraukensis* mosquitoes that were aspirated while biting man or horse [4]. The mosquitoes were collected during an arbovirus isolation program at the Mitchell River Mission, Northern Queensland (now known as Kowanyama). Following isolation, serological analysis of MAPV showed no relationship to any other viruses isolated in Queensland, with further studies ruling out a relationship to a wide range of other arboviruses known at the time [4]. Virus neutralisation studies suggest that the virus may infect a number of hosts, with highest antibody prevalence found in kangaroos and wallabies, and also in various domestic animals [5]. MPKV was isolated from *Aedes funereus* mosquitoes trapped near Maprik, New Guinea in 1966 as part of a sentinel surveillance program. Serological analysis demonstrated a relationship to MAPV and TRUV via complement fixation tests [6]. Antibody to MPKV has been detected in sera of sentinel ruminants and pigs collected in the Northern Territory between 1985 and 1997 [7]. TRUV and GGV are believed to belong to this serogroup based on antigenic comparisons [8], however no genetic data exists to confirm this. TRUV and GGV were isolated in Australia in 1965 and 1970 respectively [9, 10]. A third virus was also characterised in this study; Buffalo Creek virus (BUCV; isolate DPP0186), which was isolated from *Anopheles meraukensis* mosquitoes in Darwin, NT in 1982 as part of a mosquito surveillance trapping program established in the Northern Territory to isolate viruses. Indirect immunofluorescence and neutralisation tests subsequently showed BUCV to be a serologically distinct virus [7]. Neutralising antibodies have been detected in cattle, pig and human sera samples.

Despite the association with human illness, there has been no genetic sequencing of the Mapputta group viruses previous to this study. The genome sequencing and analysis of MAPV, MPKV and BUCV in this study indicate that all three viruses are related to each other and form a distinct group within the genus *Orthobunyavirus*.

## Materials and Methods

### Virus culture and RNA extraction

**Figure 1:**
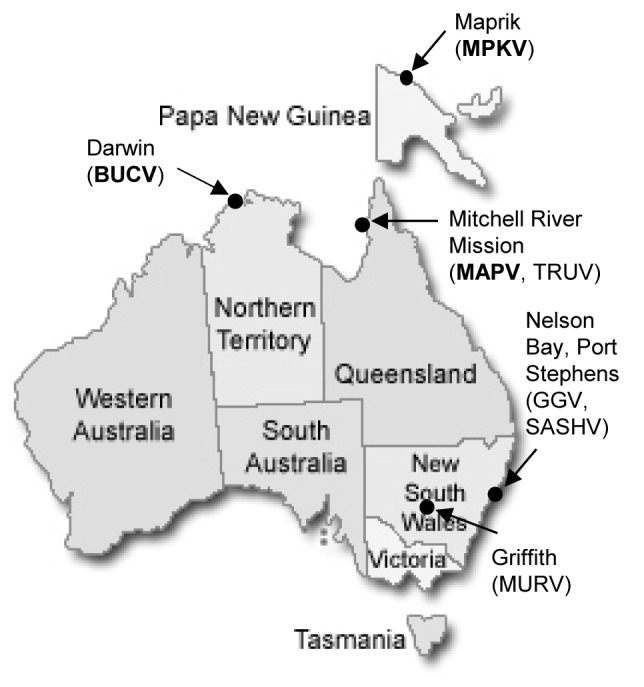
Map of Australia and Papua New Guinea showing geographical locations of the first isolations of MAPV, MPKV and BUCV. MAPV was isolated in 1960 from *Anopheles meraukensis* mosquitoes near the Mitchell River Mission in Queensland and MPKV was isolated in 1966 from *Aedes funereus* mosquitoes at the Southern foothills of the Prince Alexander Range near Maprik, Papua New Guinea. BUCV was isolated from *Anopheles meraukensis* mosquitoes in Darwin, Northern Territory in 1982. First isolation locations of TRUV, GGV, SASHV and MURBV are shown for comparison.

MAPV (isolate MRM186), MPKV (isolate MK7532) and BUCV (isolate DPP186) were obtained from the Berrimah Veterinary Laboratories, Darwin, NT, Australia (Fig. 1, Table 1). Viruses were propagated in BSR cells (a subclone of the baby hamster kidney BHK-21 cell line) grown in supplemented Basal Medium Eagle (Gibco) at 37^o^C and harvested, approximately three to four days post infection, when the first signs of cytopathic effect (CPE) were observed, as previously described [11]. The infected cell culture supernatant was collected, centrifuged at 1600 × g for 10 min to remove cell debris, and the viral pellet was subsequently obtained by ultracentrifugation at 70 000 × g for 1 hr using a Beckman 70Ti rotor. The pellet was resuspended in Buffer RLT (Qiagen) containing β-mercaptoethanol and total RNA extracted from the crude virus pellet using the RNeasy Mini Kit (Qiagen) according to manufacturer’s specifications.

**Table 1:** Isolation and seroprevalence information for Mapputta, Maprik and Buffalo Creek viruses.

**Virus**	**Isolated from**	**Isolate number**	**Year isolated**	**Location**	Neutralising antibodies*
**Mapputta (MAPV)**	*Anopheles meraukensis*	MRM186	1960 (QIMR)	Mitchell River Mission, Queensland	Man (2/470), cattle(18/96), sheep (1/38), horses (2/50), pigs (2/36), kangaroos (16/74), wallabies (23/70)and rats (3/84), domestic fowl (1/99)
**Maprik (MPKV)**	*Aedes funereus* complex	MK 7532	1966 (ANU)	Maprik, New Guinea	cattle (6/1925), pig (1/423) and buffalo (1/594)
**Buffalo Creek (BUCV)**	*Anopheles meraukensis*	DPP186	1982 (BVL)	Darwin, NT	Cattle (19/2144), pig (1/423), human (1/244)

QIMR, Queensland Institute of Medical Research; ANU, Australian National University; BVL, Berrimah Veterinary Laboratory.

*Previously published data [5, 7].

### Sequencing of complete viral genomes

The sequencing of BUCV was performed using the PCR-select cDNA Subtraction Kit (Clontech) as previously described [11], with the exception that Akabane virus (AKAV) was used as the driver in the reaction. The genome fragments generated were cloned into pCR-Blunt II TOPO and sequenced using traditional Sanger sequencing on a Genetic Analyser 3130*xl* (Applied Biosystems). Primary assembly of data and generation of consensus sequences was performed using SeqMan Pro v. 8.0.2 (Lasergene v. 8 DNASTAR). The generated sequence was subsequently used to design PCR primers (available on request) for sequence confirmation and to fill the gaps between contigs.

Total RNA from MAPV and MPKV was converted to double stranded cDNA using the Superscript ds cDNA synthesis kit (Invitrogen) and 100 pmol random hexamer according to manufacturer’s instructions. The cDNA material was prepared for high-throughput sequencing using the TruSeq (Illumina) protocols and standard multiplex adaptors. A paired-end, 100- or 150-base-read protocol was used for sequencing on an Illumina GAIIx instrument, at Micromon, Monash University, Clayton, Victoria as previously described [12]. Primary assembly of raw data and generation of consensus sequences were performed using the programs Velvet 1.1.04 [13], Geneious Pro 5.4 (Biomatters), Artemis [14] and CLC Genomics Workbench (CLC bio). Routine sequence management and the design of PCR primers was performed using the programs SeqMan Pro v. 8.0.2 (Lasergene v. 8 DNASTAR), CloneManager v. 9 (Sci Ed Central) and Sequencher 5.0 (Gene Codes Corporation).

### Confirmatory PCRs

Confirmatory PCRs were performed to fill gaps between the contigs assembled above, and to confirm regions of low sequence coverage (less than 15 × coverage). Total RNA was extracted from 100 µl of virus-infected cell culture supernatant using the RNeasy Mini Kit and was converted to single stranded cDNA using random hexamers and the Superscript III Kit (Invitrogen), to be used as template for the PCR. PCR primer pairs (Geneworks) were designed using the genome sequences assembled above, each amplifying 500–800 nt regions spanning areas requiring completion or confirmation. The subsequent PCR products were sequenced on a Genetic Analyser 3130*xl* using the Big-Dye Terminator kit (Applied Biosystems), and a contiguous consensus sequence generated for each of the three viral genome segments (excluding the genome termini) using Sequencher.

### Rapid amplification of cDNA ends

To determine the sequence of the 5’and 3’genome termini of the viruses, a modified protocol for the rapid amplification of cDNA ends (RACE) using ligated cordecypin-blocked adaptors was used as previously described [11]. To increase specificity, in this instance, RACE PCRs were performed using a modified adaptor specific primer with the sequence 5’-AACGCCATTTCCACCTTCTCTTC**AGTAG**—3’ which included 2 or 5 additional nucleotides (underlined or in bold respectively) specific to the conserved genome termini of orthobunyaviruses, and virus specific primers. The resulting PCR products were cloned into pCR2.1-TOPO (Invitrogen) and the cloned fragments were subsequently sequenced using vector specific primers on the Genetic Analyser 3130*xl*. In addition, some RACE PCR products were sequenced directly using the Illumina platform as described above.

### Predictive protein analysis

Analysis of deduced proteins was performed using PredictProtein for prediction of topology [15], and SignalP [16] for presence of signal peptides. Predicted glycosylation sites were determined using the NetNGlyc 1.0 Server (http://www.cbs.dtu.dk/services/NetNGlyc/). Pairwise amino acid and nucleotide sequence identity of the ORFs of each segment and the deduced protein was calculated using the Needleman-Wunsch algorithm with the EBLOSUM62 or EDNAFULL matrix respectively, implemented in the European Molecular Biology Open Source software unit (EMBOSS) (http://www.ebi.ac.uk/Tools/psa/emboss_needle/) [17].

### Phylogenetic analysis

Phylogenetic trees were constructed using 30 complete L protein sequences, 46 complete Gn/NSm/Gc polyprotein sequences, and 51 complete N protein sequences, of orthobunyaviruses accessed from GenBank and the appropriate MAPV, MPKV and BUCV protein sequences. Tomato spotted wilt virus (TSWV), a member of the genus *Tospovirus*, was included as an out-group. Additional Bayesian trees were constructed for each segment using the protein sequences for MAPV, MPKV, BUCV and 14 selected viruses representing all five genera of family *Bunyaviridae*. Amino acid sequences were aligned using the MUSCLE 3.6 algorithm [18]. Maximum likelihood (ML) trees were constructed using MEGA5 [19], employing the WAG model of aa substitution with a gamma distribution of rate variation and 1000 bootstrap replications. Bayesian analyses of aa sequence alignments were performed with BEAST software [20], using a WAG model of aa substitution with gamma+invariant site heterogeneity. A lognormal relaxed clock model was also used, with a tree prior set to coalescent:exponential growth. The model was run with a MCMC chain length of 10,000,000 with the output logged every 1000 steps producing 10,000 trees. The maximum clade credibility tree was chosen using Tree Annotator (1000 tree burn-in) and trees were created using FigTree v1.4 (http://tree.bio.ed.ac.uk/software/figtree/).

### Serology

Hyperimmune mouse serum was prepared to each bunyavirus as previously described [7, 21.]. Serological cross-reactivity between viruses was assessed using the virus neutralization test. Viruses were grown in BSR cells, diluted and titrated to give a titre of 100 TCID_50_ in a volume of 50 µl. Antibody to each virus was serially diluted two-fold from 1:2 to 1:4096 prior to the addition of virus. The virus (50 µl) and antibody (50 µl) mix was incubated at 37^o^C for one to two hours before 100 µl of a 2 × 10^5^ cell suspension was added. Each test was repeated in quadruplicate and the endpoint was determined according to the Reed and Muench method where 50% of the wells showed CPE at five days post inoculation [22].

## Results and Discussion

### Complete genome sequencing

High throughput sequencing (HTS) using the Illumina platform provided a superior approach to the traditional PCR-select cDNA subtraction method and Sanger sequencing previously used for obtaining novel viral sequences. The HTS method was considerably less laborious and provided more data and greater coverage of the genome. Therefore, following the sequencing of the BUCV genome using the traditional method, HTS was adopted as the method of choice for sequencing the MAPV and MPKV genomes.

The PCR-select cDNA subtraction method and traditional Sanger sequencing used to obtain sequence data for BUCV yielded approximately 83% coverage of the genome. HTS produced higher genome coverage of 97% and 91% for MAPV and MPKV, respectively. Sequencing of low coverage regions, gaps between contigs, and genome termini for the three viruses was performed using PCR and the RACE technique, with or without cloning, and using traditional Sanger sequencing. In addition, several of the genome termini (produced using the RACE technique) were opportunistically sequenced using the Illumina platform. The HTS approach for the sequencing of RACE products proved to be a highly effective method for obtaining genome ends, and eliminated the need for laborious sample preparation techniques such as cloning.

Several ambiguities resulting in amino acid transitions were observed in the nucleotide sequence of MPKV and BUCV M and L segments. Despite multiple sequencing events using different preparations of template, they could not be resolved and are noted in the respective GenBank entries. We speculate that these conflicts in sequencing could be representative of a mixed population of viruses or as a result of spontaneous mutations occurring during passaging of the viruses in cell culture.

The genetic arrangement and size of the genome segments for MAPV, MPKV and BUCV (Table 2) are similar to those of other viruses of the genus *Orthobunyavirus*. Nucleotide and amino acid sequence identities of MAPV, MPKV, BUCV and other representative orthobunyaviruses are shown in Table 3. The complete genome sequences of MAPV, MPKV and BUCV are listed in the GenBank database (accession numbers KJ481921–3, KJ481924–6 and KJ481927–9 respectively).

**Table 2:** Genome segments of MAPV, MPKV and BUCV.

		**Length (nt/aa) for indicated virus**				
**Segment**	**Region**	**MAPV**	**MPKV**	**BUCV**	**MURBV**	**SASHV**
S	3’ UTR	38	50	44	33*	34*
	N ORF	711 / 236	711 / 236	714 / 237	714 / 237	714 / 237
	5’ UTR	118	150	169	65*	44*
	**Segment total**	**867**	**911**	**927**	812*	792*
M	3’ UTR	32	46	40	31*	N/A
	M Polyprotein ORF	4113 / 1370	4140 / 1379	4116 / 1371	4116 / 1371	4131/1377
	5’ UTR	211	173	182	179*	N/A
	**Segment total**	**4356**	**4359**	**4338**	4326*	4131*
L	3’ UTR	42	50	47	45*	43*
	L ORF	6726 / 2241	6720 / 2239	6729 / 2242	6729 / 2242	6637*/2212*
	5’ UTR	119	73	145	146	N/A
	**Segment total**	**6887**	**6843**	**6921**	6920*	6680*

Murrumbidgee virus (MURBV, GenBank accession numbers KF234253–5) and Salt Ash virus (SASHV, GenBank accession numbers KF234256–8) have been included for comparison.

N/A: Not available;

*incomplete genome sequence.

**Table 3:** Nucleotide and amino acid identity comparisons for the Mapputta Group viruses and representative orthobunyaviruses.

		**MAPV**	**MPKV**	**BUCV**	**MURBV**	**SASHV**	**BUNV**	**WYOV**	**OROV**	**LACV**	**JCV**
**MAPV**	**S**	-	72.0	67.8	67.8	67.9	37.2	37.8	37.1	42.7	44.2
	**M**	-	46.9	49.2	49.7	47.7	32.1	31.7	29.8	31.8	32.4
**L**	-	62.8	59.5	59.4	61.8	49.4	49.7	46.0	50.9	51.5
**MPKV**	**S**	67.9	-	70.0	70.5	87.3	36.5	38.4	36.4	44.5	44.5
	**M**	58.0	-	48.7	48.6	61.2	33.6	31.9	30.6	33.6	32.4
	**L**	65.9	-	61.5	61.4	86.6	49.4	49.4	48.3	50.8	51.1
**BUCV**	**S**	62.8	67.5	-	98.3	68.5	36.7	39.5	38.7	41.4	39.4
	**M**	59.3	59.2	-	98.3	49.3	34.0	34.0	30.4	34.3	33.2
	**L**	63.7	64.7	-	99.0	59.9	48.6	48.6	46.8	50.1	50.2
**MURBV**	**S**	63.9	68.6	95.8	-	67.6	36.3	38.7	38.6	41.4	39.4
	**M**	58.6	58.6	95.9	-	49.2	33.9	33.9	30.3	34.6	33.2
	**L**	63.7	65	95.9	-	60.0	48.7	48.7	46.9	50.1	50.1
**SASHV**	**S**	68.8	81.7	67.9	67.7	-	35.2	38.5	38.1	43.9	43.4
	**M**	58.7	64.0	57.7	57.5	-	34.8	34.1	32.4	35.7	34.4
	**L**	64.0	76.3	64.1	64.0	-	49.2	49.8	47.4	50.9	50.5
**BUNV**	**S**	50.5	51.8	52.2	52.8	51.4	-	62.7	41.5	43.0	44.3
	**M**	50.8	49.6	50.2	51.0	51.6	-	51.2	34.0	43.1	42.5
	**L**	57.5	57.7	57.9	58.1	58.3	-	67.0	50.4	56.0	55.1
**WYOV**	**S**	50.6	53.7	53.8	53.4	55.9	64.7	-	40.8	48.5	46.8
	**M**	52.6	53.0	51.9	52.2	52.7	59.0	-	33.6	40.8	40.4
	**L**	58.1	58.2	58.7	58.8	57.6	67.0	-	49.2	55.2	55.2
**OROV**	**S**	48.2	48.2	53.1	51.5	49.5	52.6	49.8	-	41.7	43.0
	**M**	49.6	49.9	50.3	49.7	49.6	50.0	49.5	-	32.1	32.2
**L**	57.3	56.3	57.1	57.2	57.3	57.9	57.5	-	51.9	51.6
**LACV**	**S**	55.6	54.5	51.5	52.6	54.8	55.4	55.7	56.6	-	82.1
	**M**	48.8	50.4	49.3	49.0	51.3	54.5	54.2	50.5	-	72.2
	**L**	58.5	58	58.0	57.9	58.4	59.1	59.9	58.4	-	83.5
**JCV**	**S**	53.8	52.3	50.7	47.6	53.1	56.2	52.2	56.1	79.2	-
	**M**	50.2	51.5	50.7	50.6	52.3	54.8	55.1	51.2	68.8	-
	**L**	59.3	59.4	58.9	59.2	58.6	60.4	60.7	59.2	74.2	-

Nucleotide (bottom left) and amino acid (top right) identity values of sequences from the complete N ORF (S segment), polyprotein ORF (M segment) and RdRp ORF (L segment) of the Mapputta group viruses. Viruses of the Bunyamwera (Bunyamwera (BUNV), Wyeomyia (WYOV)), Simbu (Oropouche, OROV) and California (LaCrosse, (LACV), Jamestown Canyon (JCV) serogroups are also included for comparison.

### Untranslated regions and terminal end sequences

**Figure 2:**
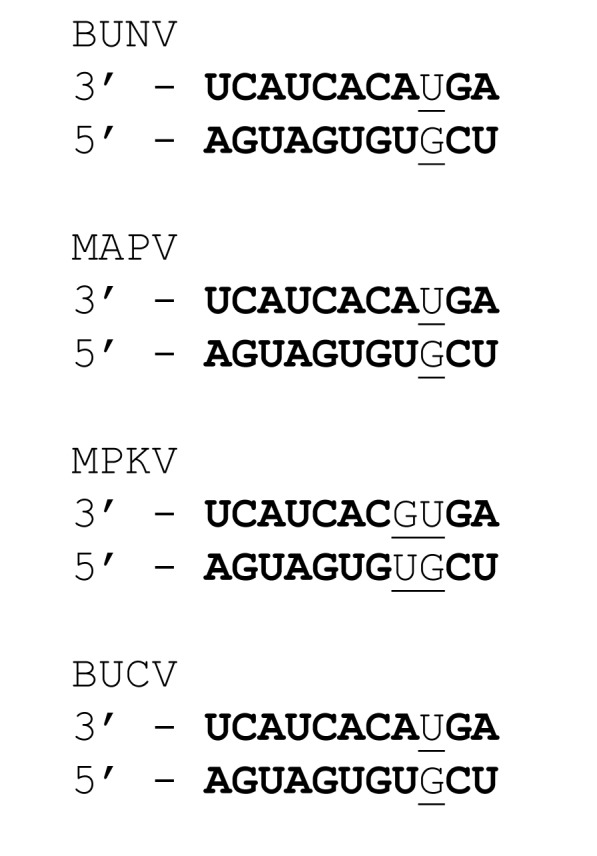
Genome terminal sequences of MAPV, MPKV and BUCV compared with the orthobunyavirus type species, BUNV. MAPV and BUCV have the universally conserved complementary terminal 11 bases (in bold) typically observed in orthobunyaviruses, including a non-canonical base pairing at position 9 (underlined). MPKV differs from the consensus sequence with an additional non-canonical base pairing at position 8 (underlined).

The 3’ and 5’ untranslated regions (UTR) of the genome segments of bunyaviruses contain signals for transcription and replication, as well as encapsidation of genomic and antigenomic RNAs by the N protein [23–27]. Complementarity of the terminal 15–16 nucleotides of each segment enables the formation of panhandles, thus providing a distinction between viral and non-viral RNA [28]. This level of complementarity of the terminal end sequences is evident in the genome segments of MAPV, MPKV and BUCV. Orthobunyaviruses typically contain a highly conserved stretch of 11 invertedly complementary nucleotides at the 3’ and 5’ termini. This conserved sequence usually includes a non-canonical base pair (U-G) at position 9. This consensus sequence was observed in MAPV and BUCV, however, the MPKV genome segments have an additional non-canonical base pair at position 8 (Fig. 2). A deviation from the consensus sequence has previously been reported for Akabane virus (AKAV), where an additional mismatch similarly occurs at position 8 [29, 30]. This supports the suggestion by Elliott and Blakqori [2] that variations in the termini could be more extensive than currently appreciated, considering that many sequences have been determined using biased oligonucleotide primers designed to the terminal consensus sequences derived from the first genomes sequenced. Often viruses within a serogroup contain three or four additional conserved nucleotides adjacent to the 11-nt terminal sequence. In MAPV, MPKV and BUCV, four conserved nt are observed in the M and L segments, and three conserved nt in the S segment adjacent to the 11-nt terminal sequence.

### S segment—Nucleoprotein

The S segment encodes the nucleoprotein (N), which encapsidates the viral genomic RNA segments to form ribonucleoprotein (RNP) complexes. These complexes interact with the viral L polymerase and glycoproteins, and thus have an important role in RNA transcription, replication, and virion stability. BLAST database searches of the putative N proteins of MAPV (236 aa), MPKV (236 aa) and BUCV (237 aa) indicate highest identity to the N proteins of the recently identified orthobunyaviruses Murrumbidgee virus (MURBV) (MAPV, 68%; MPKV, 70%; BUCV, 98%) and Salt Ash virus (SASHV) (MAPV, 68%; MPKV, 87%; BUCV, 68%) [31] suggesting that MURBV and SASHV likely also belong to the Mapputta group. The ICTV demarcation criteria for virus species within the genus *Orthobunyavirus* states that the aa sequence of the N protein, where known, differ by more than 10% [1]. Therefore, according to this criteria, BUCV and MURBV are likely the same species as the amino acid sequence of the N proteins differ by less than 2%. Pairwise sequence identity of the nucleoprotein sequences of MAPV, MPKV and BUCV, and other selected orthobunyaviruses, were conducted and are shown in Table 3. The three Mapputta group viruses exhibit a high level of identity (63–68% nt and 68–72% aa). The level of identity these viruses share with viruses of other serogroups (48–56% nt, 35–45% aa) and is comparable to other inter-serogroup comparisons (50–57% nt, 41–49% aa) within the genus *Orthobunyavirus*.

The N proteins of the four major serogroups (Bunyamwera, California, Group C and Simbu) within the genus *Orthobunyavirus* display global conservation of 46 residues with another 14 residues conserved amongst 90% of these viruses [32]. As observed in Leanyer virus [33] (also isolated in Australia), these amino acids are likewise not strictly conserved in MAPV, MPKV and BUCV. Of the 60 residues conserved amongst 90% of the viruses, 45 (MPKV, BUCV) and 44 (MAPV) are conserved. Nonetheless, the conservation of 4 residues with an identified role in RNP packaging, and 9 out of 10 residues identified as being important in RNA synthesis [32], is observed in each of the reported viruses.

Whilst most orthobunyaviruses also encode the smaller NSs protein in an overlapping reading frame [34–37], a feature of MAPV, MPKV and BUCV is the absence of this ORF. The NSs ORF is also absent from orthobunyaviruses of the Anopheles A, Anopheles B and Tete serogroups [38], and is severely truncated in orthobunyaviruses of the Wyeomyia group [39]. Although not essential, the NSs protein’s primary role is in modulating the host-cell antiviral response by acting as an antagonist of the interferon system [40, 41]. BUNV engineered with an NSs deletion was shown to be attenuated in IFN competent mice [42], however, Tacaiuma virus (TCMV), which is associated with a febrile illness in humans, appears to have the ability to overcome the host innate immune response despite lacking the NSs protein [38]. A similar mechanism that allows TCMV to overcome the human defences may be present in MAPV, MPKV, BUCV and, presuming they too lack the NSs genes, GGV and TRUV (noting that BUCV, GGV and TRUV have presumed links with human disease). The N proteins in viruses of the Anopheles A, Anopheles B and Tete serogroups are longer than most orthobunyavirus N proteins but this phenomenon is not seen in the NSs-lacking Wyeomyia group or Mapputta group viruses reported here.

### M segment—polyprotein

The M segment of orthobunyaviruses encodes a polyprotein, which is cleaved post-translationally into two glycoproteins, Gn and Gc, and the non-structural NSm protein. The M segment of MAPV, MPKV and BUCV putatively encodes a polyprotein consistent with those found in other orthobunyaviruses. BLAST database searches of the deduced 1370-aa, 1379-aa and 1371-aa polyproteins indicate highest identity to the polyproteins of MURBV (MAPV, 49%; MPKV, 48%; BUCV, 98%) and SASHV (MAPV, 47%; MPKV, 61%; BUCV, 49%) Pairwise sequence identities of 58–59% (nt) and 47–49% (aa) are observed between the M polyproteins of MAPV, MPKV and BUCV (Table 3). The level of identity these viruses share with viruses of other serogroups of the genus (49–53% nt, 30–36% aa) is comparable to other inter-serogroup comparisons (50–55% nt and 32–43% aa).

SignalP analysis predicted cleavage sites at residues 13, 16 and 15 for MAPV, MPKV and BUCV, respectively. As for other orthobunyaviruses [29, 33, 43, 44], the cleavage site between Gn and NSm is predicted to occur after a conserved arginine residue for MAPV (R_301_), BUCV (R_301_) and MPKV (R_304_). The junction between the NSm and Gc in orthobunyaviruses is not always well conserved [33, 44], and similarly cleavage sites could not be clearly identified in our viruses. SignalP analysis predicts a cleavage site at VKA_469_-EV for MPKV, consistent with a typical signalase site, and two sites are predicted for MAPV (IIS_460_-TR and TRG_463_-AN) and BUCV (SFA_463_-IA or AIA_465_-TP). Transmembrane regions identified by analysis with PredictProtein follow a similar pattern for all three viruses, with six regions identified. Two regions are in the Gn (range 204–231 and 233–253), three in the NSm (range 310–331, 358–381, and 443–467) and one in the Gc (range 1324–1355). These results are consistent with predictions of other bunyaviruses [33, 44, 45].

**Figure 3:**
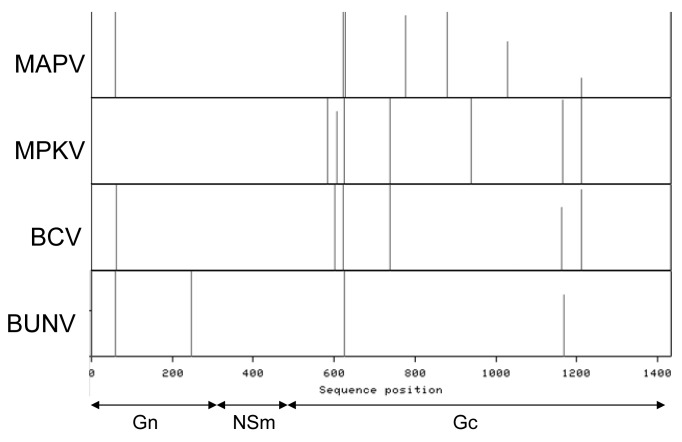
Predicted glycosylation sites in the MAPV, MPKV and BUCV M polyproteins. The likelihood of glycosylation at site N_903_ in MPKV is low due to the presence of a proline residue after the arginine residue. Predicted sites in BUNV have been included for comparison.

Seven glycosylation sites are predicted in MAPV (one in Gn, six in Gc) and MPKV (all in Gc), and six sites are predicted in BUCV (one in Gn, five in Gc) (Fig. 3). MAPV has at least three potential sites in Gc that are unique, whilst MPKV has one. Five of the sites in MPKV and BUCV are conserved between the two viruses. Interestingly there is no glycosylation site predicted in the MPKV Gn protein, which is highly atypical of bunyaviruses. This was confirmed by repeated sequencing using different template preparations. A BUNV reverse genetics approach demonstrated that glycosylation of N_60_ in the Gn is essential for correct protein folding of both Gn and Gc proteins and therefore affects the viability of the virus [46]. An analogous glycosylation site is present in the MAPV and BUCV Gn proteins. It is unclear why the MPKV Gn protein does not contain any glycosylation sites; perhaps it is a consequence of passage in cell culture, however, it is clearly not essential for virus survivability in cell culture. Only two glycosylation sites are conserved in all three viruses, both situated in the Gc protein. The first of these correspond to the highly conserved BUNV N_624_ site present in viruses of the bunyamwera serogroup. The second is conserved with that of AKAV but none others, when compared to viruses from Simbu, California and Bunyamwera serogroups.

### L segment—RNA-dependent RNA polymerase

The L segment of bunyaviruses encodes the RNA dependent RNA polymerase (RdRp) responsible for replication and transcription of the viral RNA. BLAST database searches of the respective deduced 2241-aa, 2239-aa and 2242-aa L proteins indicate highest identity to the L proteins of MURBV (MAPV, 60%; MPKV, 62%; BUCV, 99%) and SASHV (MAPV, 63%; MPKV, 88%; BUCV, 61%). Pairwise sequence identities of 64–66% (nt) and 60–63% (aa) are observed between the L proteins of MAPV, MPKV and BUCV (Table 3). The level of identity these viruses share with viruses of other serogroups from the genus (56–59% nt, 46–52% aa) is comparable to other inter-serogroup comparisons (58–61% nt and 51–56% aa).

RdRps contain regions that are highly conserved amongst viruses within the family and indeed amongst the negative sense RNA viruses, reflective of the universal functions of this protein [47–49]. These regions are highly conserved in MAPV, MPKV and BUCV polymerases. For example, all three viruses contain conserved Regions I and II centred around the highly conserved dipeptides PD and RY typically observed in bunyaviruses and arenaviruses [48]. Similarly, high conservation of premotif A and motifs A-E are also apparent in all three viruses, including the almost invariant amino acids seen in all RdRps [47, 49] (S1 Fig.).

### Phylogenetic analysis

**Figure 4:**
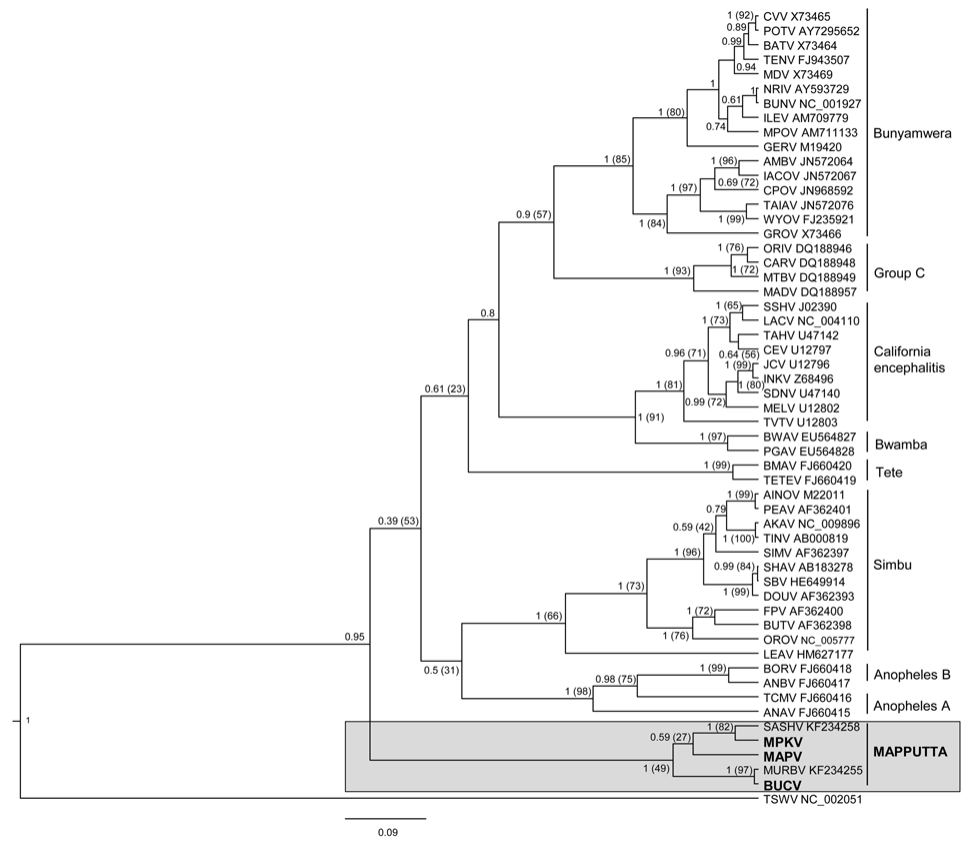
Phylogenetic relationship of the nucleocapsid protein of the Mapputta Group viruses and other selected orthobunyaviruses. Relationship was inferred by Bayesian analysis of the protein sequence alignment. A WAG model of aa substitution with gamma+invariant site heterogeneity was used. Numbers represent Bayesian posterior probabilities (Maximum Likelihood Bootstrap values). Tomato spotted wilt virus (TSWV) has been included as an out-group. Tree is drawn to scale measured in substitutions/site as indicated by the scale bar.

**Figure 5:**
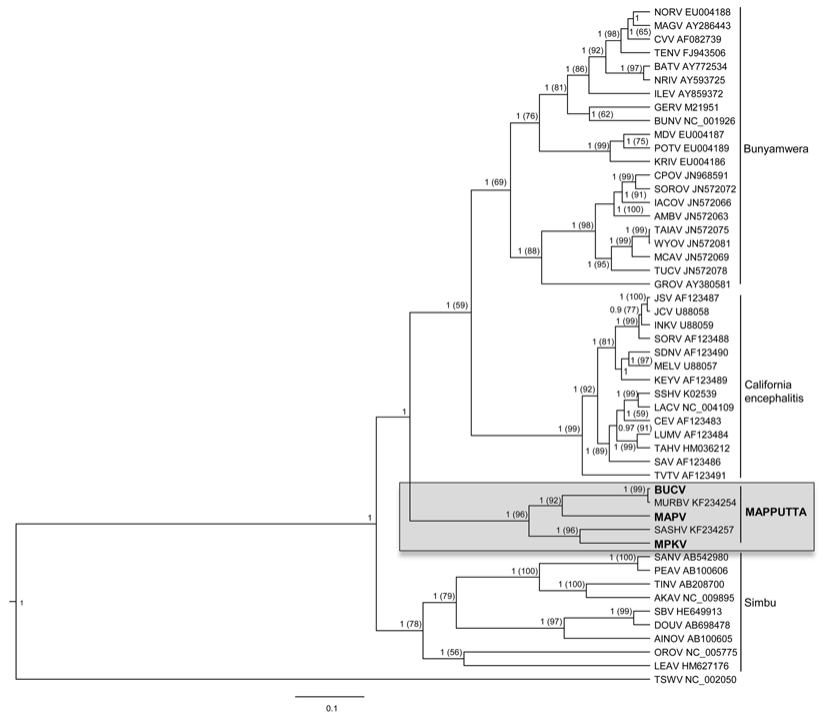
Phylogenetic relationship of the M segment polyprotein of the Mapputta Group viruses and other selected orthobunyaviruses. Relationship was inferred by Bayesian analysis of the protein sequence alignment. A WAG model of aa substitution with gamma+invariant site heterogeneity was used. Numbers represent Bayesian posterior probabilities (Maximum Likelihood Bootstrap values). Tomato spotted wilt virus (TSWV) has been included as an out-group. Tree is drawn to scale measured in substitutions/site as indicated by the scale bar.

**Figure 6:**
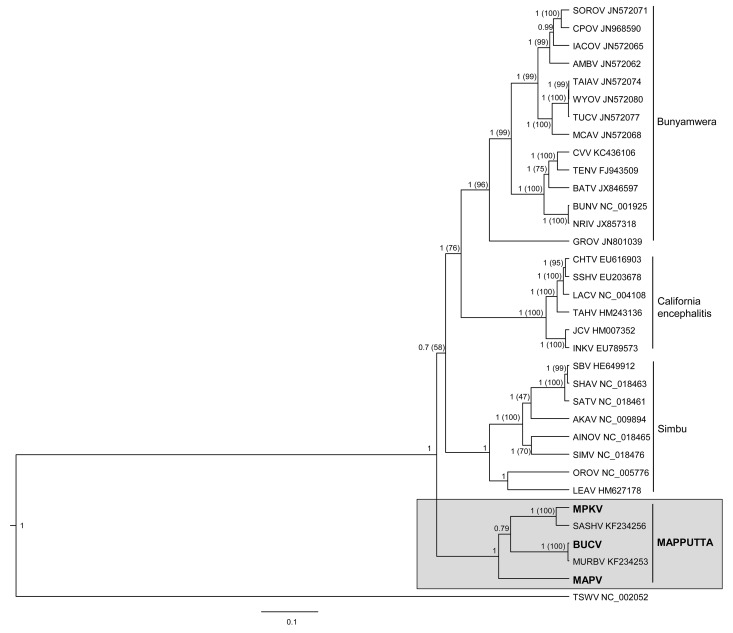
Phylogenetic relationship of the polymerase protein of the Mapputta Group viruses and other selected orthobunyaviruses. Relationship was inferred by Bayesian analysis of the protein sequence alignment. A WAG model of aa substitution with gamma+invariant site heterogeneity was used. Numbers represent Bayesian posterior probabilities (Maximum Likelihood Bootstrap values). Tomato spotted wilt virus (TSWV) has been included as an out-group. Tree is drawn to scale measured in substitutions/site as indicated by the scale bar.

Bayesian (Figs. 4–6) and Maximum Likelihood (ML) (data not shown) phylogenetic analyses of the translated sequences of the nucleocapsid, polyprotein and RdRp show similar topologies. It is evident that MAPV, MPKV and BUCV share a monophyletic origin with the recently described SASHV and MURBV. All five viruses belong to the genus *Orthobunyavirus*, but are clearly distinct from all orthobunyavirus serogroups described to date. The Bayesian trees (Figs. 4–6) demonstrate with high confidence that MPKV forms a clade with SASHV, and BUCV forms a clade with MURBV. The phylogenetic trees suggest that the Mapputta group was one of the earliest diverged groups within the genus, which is most prominently demonstrated by the polymerase and the nucleocapsid (Figs. 4 and 6). Due to this high divergence from other groups within the genus, additional Bayesian trees were constructed using representative viruses from all other genera of the family *Bunyaviridae* to demonstrate that the Mapputta group belongs to the genus *Orthobunyavirus* (S2 Fig.). These smaller trees, however, do not accurately predict the exact placement of the Mapputta group in relation to the other groups within the genus. This is due to the inability to effectively align and analyse a complete gamete of orthobunyaviruses with viruses from the other four highly diverged genera. For this reason, a more accurate portrayal of the relationship of the Mapputta group within the genus is depicted in Figs. 4–6. Speculatively, the distant and ancestral relationship of the Mapputta group to the other groups within the genus could be indicative of a long co-existence within a unique ecosystem and a unique host. This theory is supported by the previously published observations (Table 1) that Australian marsupials (kangaroos and wallabies) may be a key host species of these viruses.

Interestingly, the intra-group pairing of BUCV, MPKV and MAPV is not consistent across the different proteins analysed. Bayesian and ML analysis of the polyprotein suggests that BUCV forms a clade with MAPV, while analysis of the nucleoprotein suggests that MAPV in fact clusters more closely with MPKV. Analysis of the polymerase however, is less conclusive; Bayesian analysis suggests that BUCV forms a clade with MPKV (0.79 posterior support) and ML (data not shown) suggests that MAPV forms a clade with MPKV (bootstrap support 78). The lack of clarity of pairings within the group may be resolved once sequence is available for other related viruses such as GGV and TRUV. Furthermore, reassortment plays a fundamental role in bunyavirus evolution [50], thus it may have had a role in the currently perceived relationships within this group.

### Serology

Serological cross-neutralisation tests were performed with all five presumed Mapputta group viruses including GGV and TRUV. Antibodies generated to all five viruses were able to neutralise all the other viruses in the group to varying levels (Table 4). MAPV and MPKV antibodies effectively neutralised all of the known Mapputta group viruses. Whilst antibodies generated to BUCV, GGV and TRUV also neutralised all of the Mapputta group viruses, they neutralised MPKV at only trace (1:4 dilution) level. The cross reactivity results indicate that a positive clinical serological result could be indicative of infection with any of the viruses in the Mapputta group, and this must be considered in any future serological surveys aiming to identify new hosts or links with disease.

**Table 4:** Cross neutralisation of viruses of the Mapputta group.

		**Antibody**				
		**MAPV**	**MPKV**	**BUCV**	**GGV**	**TRUV**
	MAPV	512	64	64	16	16
	MPKV	8	256	4	4	4
**Antigen**	BUCV	16	16	256	16	128
	GGV	16	64	32	2048	8
	TRUV	16	16	64	16	256

## Concluding Remarks

The complete genomes of MAPV, MPKV and BUCV have been sequenced and their analysis demonstrates a case for inclusion within the genus *Orthobunyavirus* in the family *Bunyaviridae*. All three viruses exhibit a similar genetic structure including the absence of the NSs gene, which has been observed in only a few other orthobunyaviruses. MPKV in particular demonstrates two characteristics that are highly unusual for orthobunyaviruses. The first is the deviation from the conserved bunyavirus terminal sequence at position 8 of the 3’ genomic RNA on all three genome segments of MPKV. The second is the seemingly non-glycosylated Gn protein, which in other bunyaviruses typically contains at least one universally conserved site with an essential role in replication.

Comparisons of MAPV, MPKV and BUCV indicate that these viruses are most closely related to MURBV and SASHV. High sequence identities of BUCV and MURBV indicate that they are the same virus species. MPKV and SASHV exhibit a high degree of identity though are two distinct viruses. The impending sequencing of TRUV and GGV is anticipated to reveal their relationship and to further shape the dynamics of the group. All of the Mapputta group viruses have been isolated from mosquito species; MAPV, BUCV, TRUV and MURBV from *Anopheles spp* and MPKV, GGV and SASHV from *Aedes spp*. The isolation areas of these viruses ranging from Maprik in Papua New Guinea to Griffith in southern NSW, Australia, indicate a large geographical area encompassing tropical to semi-arid climates. Although little is known about this group, it has importantly been implicated in human disease. GGV has been found to be involved in a disease process which mimics an acute epidemic polyarthritis-like illness [51], and evidence suggests that TRUV may also cause a similar disease [51]. In addition, a serum sample from a human diagnosed with a viral-like illness at the Royal Darwin Hospital in 1993 has tested positive to BUCV [7]. Thus, a comprehensive serological survey of the human population in these areas would be instrumental in defining this group’s role in disease.

Australia has a long history in isolating arboviruses that dates back to the 1950s [52–55]. The northern tropical regions of the country are subject to periods of high rainfall and humidity, providing ideal conditions for arthropods and arboviruses to thrive. As such, many varied arboviruses have been isolated on this continent, and a large number still remain uncharacterised. Characterisation of these ‘unknown’ viruses may be instrumental in filling the diagnostic gap left for example by the large proportion (estimated 70%) of disease with encephalitic or febrile symptoms caused by unknown agents in Australia [56, 57]. Factors such as the effects of climate change on vector distribution, global travel, and encroachment into new territories, are possibly contributing to the increasing emergence of new diseases around the world. Recently, novel bunyaviruses such as Schmallenberg virus (SBV) in Europe [58] and severe fever thrombocytopenia syndrome virus (SFTSV) in China [59] have emerged, highlighting the increasing importance of identifying and investigating new viruses that may pose a threat to human, livestock and wildlife health. New data serves to better inform and lead to the development and implementation of appropriate early detection, monitoring and intervention strategies.

## Supporting Information

S1 Fig RNA-dependent RNA polymerase motifs of representative orthobunyaviruses and the Mapputta group viruses. Conserved motifs are shown in boxes. Conserved residues are shaded and residues conserved for nearly all RNA dependent RNA polymerases of negative RNA viruses are marked with an asterisk (*).
(TIF)

S2 Fig Phylogenetic relationships of the S (a), M (b) and L (c) segments of MAPV, MPKV and BUCV and representational bunyaviruses. Relationships were inferred by Bayesian analysis of protein alignments. A WAG model of aa substitution with gamma+invariant site heterogeneity was used. Numbers represent Bayesian posterior probabilities.
(PPTX)
