# Correction: Genomic Characterisation of Three Mapputta Group Viruses, a Serogroup of Australian and Papua New Guinean Bunyaviruses Associated with Human Disease

**DOI:** 10.1371/journal.pone.0121466

**Published:** 2015-03-23

**Authors:** The PLOS ONE Staff

In Table 3, the third row and the 24^th^ row have been shifted one cell to the left. Please see the corrected Table 3 here.

**Table 3:** Nucleotide and amino acid identity comparisons for the Mapputta Group viruses and representative orthobunyaviruses.

		MAPV	MPKV	BUCV	MURBV	SASHV	BUNV	WYOV	OROV	LACV	JCV
**MAPV**	**S**	-	72.0	67.8	67.8	67.9	37.2	37.8	37.1	42.7	44.2
	**M**	-	46.9	49.2	49.7	47.7	32.1	31.7	29.8	31.8	32.4
	**L**	-	62.8	59.5	59.4	61.8	49.4	49.7	46.0	50.9	51.5
**MPKV**	**S**	67.9	-	70.0	70.5	87.3	36.5	38.4	36.4	44.5	44.5
	**M**	58.0	-	48.7	48.6	61.2	33.6	31.9	30.6	33.6	32.4
	**L**	65.9	-	61.5	61.4	86.6	49.4	49.4	48.3	50.8	51.1
**BUCV**	**S**	62.8	67.5	-	98.3	68.5	36.7	39.5	38.7	41.4	39.4
	**M**	59.3	59.2	-	98.3	49.3	34.0	34.0	30.4	34.3	33.2
	**L**	63.7	64.7	-	99.0	59.9	48.6	48.6	46.8	50.1	50.2
**MURBV**	**S**	63.9	68.6	95.8	-	67.6	36.3	38.7	38.6	41.4	39.4
	**M**	58.6	58.6	95.9	-	49.2	33.9	33.9	30.3	34.6	33.2
	**L**	63.7	65	95.9	-	60.0	48.7	48.7	46.9	50.1	50.1
**SASHV**	**S**	68.8	81.7	67.9	67.7	-	35.2	38.5	38.1	43.9	43.4
	**M**	58.7	64.0	57.7	57.5	-	34.8	34.1	32.4	35.7	34.4
	**L**	64.0	76.3	64.1	64.0	-	49.2	49.8	47.4	50.9	50.5
**BUNV**	**S**	50.5	51.8	52.2	52.8	51.4	-	62.7	41.5	43.0	44.3
	**M**	50.8	49.6	50.2	51.0	51.6	-	51.2	34.0	43.1	42.5
	**L**	57.5	57.7	57.9	58.1	58.3	-	67.0	50.4	56.0	55.1
**WYOV**	**S**	50.6	53.7	53.8	53.4	55.9	64.7	-	40.8	48.5	46.8
	**M**	52.6	53.0	51.9	52.2	52.7	59.0	-	33.6	40.8	40.4
	**L**	58.1	58.2	58.7	58.8	57.6	67.0	-	49.2	55.2	55.2
**OROV**	**S**	48.2	48.2	53.1	51.5	49.5	52.6	49.8	-	41.7	43.0
	**M**	49.6	49.9	50.3	49.7	49.6	50.0	49.5	-	32.1	32.2
	**L**	57.3	56.3	57.1	57.2	57.3	57.9	57.5	-	51.9	51.6
**LACV**	**S**	55.6	54.5	51.5	52.6	54.8	55.4	55.7	56.6	-	82.1
	**M**	48.8	50.4	49.3	49.0	51.3	54.5	54.2	50.5	-	72.2
	**L**	58.5	58	58.0	57.9	58.4	59.1	59.9	58.4	-	83.5
**JCV**	**S**	53.8	52.3	50.7	47.6	53.1	56.2	52.2	56.1	79.2	-
	**M**	50.2	51.5	50.7	50.6	52.3	54.8	55.1	51.2	68.8	-
	**L**	59.3	59.4	58.9	59.2	58.6	60.4	60.7	59.2	74.2	-

Nucleotide (bottom left) and amino acid (top right) identity values of sequences from the complete N ORF (S segment), polyprotein ORF (M segment) and RdRp ORF (L segment) of the Mapputta group viruses. Viruses of the Bunyamwera (Bunyamwera (BUNV), Wyeomyia (WYOV)), Simbu (Oropouche, OROV) and California (LaCrosse, (LACV), Jamestown Canyon (JCV) serogroups are also included for comparison.

## References

[1] Gauci PJ, McAllister J, Mitchell IR, Boyle DB, Bulach DM, Weir RP, et al (2015) Genomic Characterisation of Three Mapputta Group Viruses, a Serogroup of Australian and Papua New Guinean Bunyaviruses Associated with Human Disease. PLoS ONE 10(1): e0116561 doi: 10.1371/journal.pone.0116561 25588016 10.1371/journal.pone.0116561 PMC4294684

