# Supplementary figures and images for: Genomic Characterisation of Three Mapputta Group Viruses, a Serogroup of Australian and Papua New Guinean Bunyaviruses Associated with Human Disease
Source: PLoS One. 2015 Jan 14;10(1):e0116561. doi: 10.1371/journal.pone.0116561 (PMC4294684; doi:10.1371/journal.pone.0116561)

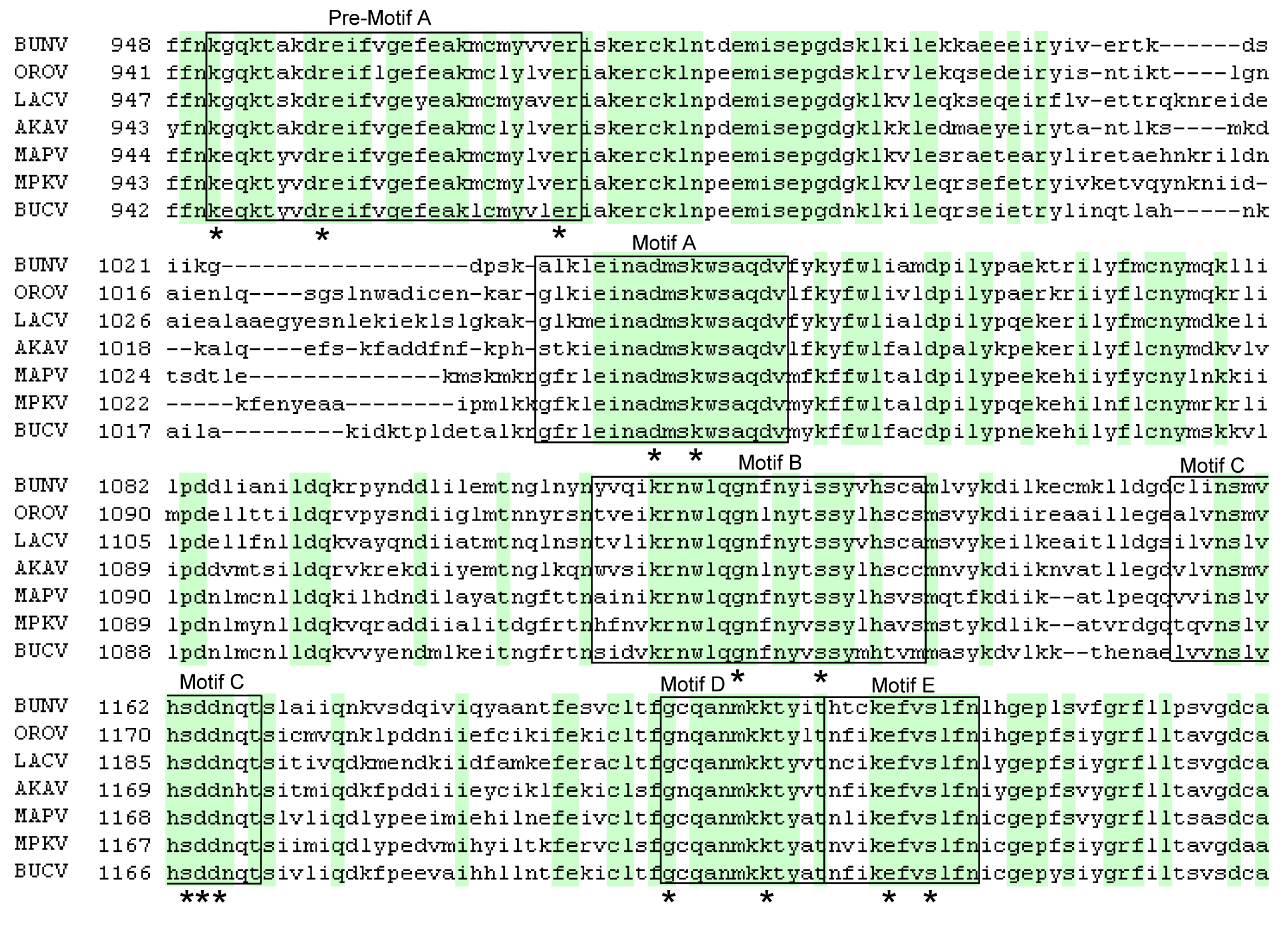

Supplement: S1 Fig — Conserved motifs are shown in boxes. Conserved residues are shaded and residues conserved for nearly all RNA dependent RNA polymerases of negative RNA viruses are marked with an asterisk (*). (TIF) [file pone.0116561.s001.tif]
