# Supplementary material for: Genomic Characterisation of Three Mapputta Group Viruses, a Serogroup of Australian and Papua New Guinean Bunyaviruses Associated with Human Disease
Source: PLoS One. 2015 Jan 14;10(1):e0116561. doi: 10.1371/journal.pone.0116561 (PMC4294684; doi:10.1371/journal.pone.0116561)
Supplement: S2 Fig — Relationships were inferred by Bayesian analysis of protein alignments. A WAG model of aa substitution with gamma+invariant site heterogeneity was used. Numbers represent Bayesian posterior probabilities. (PPTX) [file pone.0116561.s002.pptx]

## Slide 1
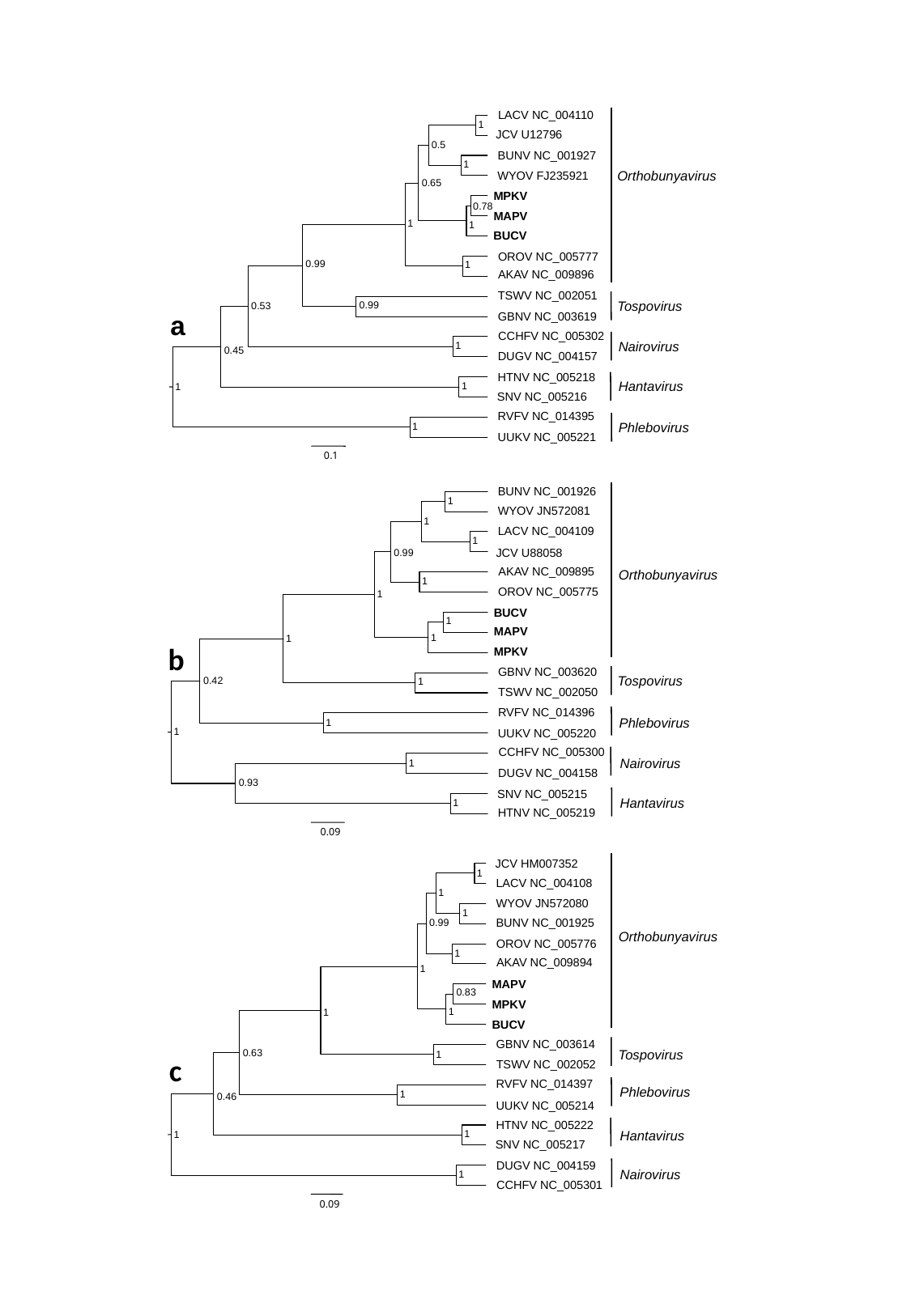

LACV NC_004110
1
JCV U12796
0.5
BUNV NC_001927
1
Orthobunyavirus
WYOV FJ235921
0.65
MPKV
0.78
MAPV
1
1
BUCV
OROV NC_005777
0.99
1
AKAV NC_009896
TSWV NC_002051
Tospovirus
0.99
0.53
a
GBNV NC_003619
CCHFV NC_005302
Nairovirus
1
0.45
DUGV NC_004157
HTNV NC_005218
Hantavirus
1
1
SNV NC_005216
RVFV NC_014395
Phlebovirus
1
UUKV NC_005221
0.1
BUNV NC_001926
1
WYOV JN572081
1
LACV NC_004109
1
JCV U88058
0.99
AKAV NC_009895
1
OROV NC_005775
1
BUCV
1
MAPV
1
1
MPKV
GBNV NC_003620
0.42
1
TSWV NC_002050
RVFV NC_014396
1
1
UUKV NC_005220
CCHFV NC_005300
1
DUGV NC_004158
0.93
SNV NC_005215
1
HTNV NC_005219
0.09
Orthobunyavirus
b
Tospovirus
Phlebovirus
Nairovirus
Hantavirus
JCV HM007352
1
LACV NC_004108
1
WYOV JN572080
1
BUNV NC_001925
0.99
OROV NC_005776
1
AKAV NC_009894
1
MAPV
0.83
MPKV
1
1
BUCV
GBNV NC_003614
0.63
1
TSWV NC_002052
RVFV NC_014397
1
0.46
UUKV NC_005214
HTNV NC_005222
1
1
SNV NC_005217
DUGV NC_004159
1
CCHFV NC_005301
0.09
Orthobunyavirus
Tospovirus
c
Phlebovirus
Hantavirus
Nairovirus
